# Dayuan Yin alleviates symptoms of HCoV-229E-induced pneumonia and modulates the Ras/Raf1/MEK/ERK pathway

**DOI:** 10.1007/s13659-024-00474-8

**Published:** 2024-11-04

**Authors:** Rui Li, Wen Zhang, Bei Huang, Guotong Sun, Yifei Xie, Junke Song, Shumei Wang, Guanhua Du

**Affiliations:** 1 Institute of Materia Medica, Chinese Academy of Medical Sciences and Peking Union Medical College, Beijing, 100050 China; 2 Guangdong Pharmaceutical University, Guangzhou, 510006 China

**Keywords:** Dayuan Yin, Viral pneumonia, HCoV-229E, Ras/Raf1/MEK/ERK

## Abstract

Viral pneumonia is characterized by inflammation in the lungs triggered by respiratory viruses. Dayuan Yin (DYY), a traditional Chinese medicine formula known for treating infectious diseases, is hypothesized to offer therapeutic benefits in treating viral pneumonia, although its specific molecular impacts remain understudied. This study aimed to evaluate the therapeutic effects of DYY in mitigating HCoV-229E virus-induced pneumonia in mice. This study employed an HCoV-229E virus-infected mouse model to investigate the therapeutic potential and underlying molecular mechanisms of DYY on virus-induced pneumonia. The respiratory function and organ indices post-treatment were assessed. Lung tissue and tracheal lesions were evaluated via immunohistochemistry. Spleen immune cell composition was analyzed using flow cytometry. Inflammatory cytokines and viral loads were quantified using hypersensitive multiplex electrochemiluminescence method and PCR analysis, respectively. The expression levels of MAS1, Ras, Raf1, MEK1/2, and ERK1/2 in lung tissues were determined through western blot analysis. DYY significantly improved respiratory function, and reduced organ pathology in infected mice. It effectively decreased viral loads and inflammatory cytokines such as IL-6, IL-1β, and TNF-α in lung tissues. Enhancements in immune response were evidenced by increased CD4/CD8 ratios in the spleen. DYY also notably upregulated MAS1 protein levels and suppressed the activation of the Ras/Raf1/MEK/ERK signaling pathway. DYY enhanced respiratory function and exerted significant antiviral and immunomodulatory effects in mice infected with the HCoV-229E virus, primarily by modulating MAS1 expression and inhibiting the Ras/Raf1/MEK/ERK pathway.

**Graphical Abstract:**

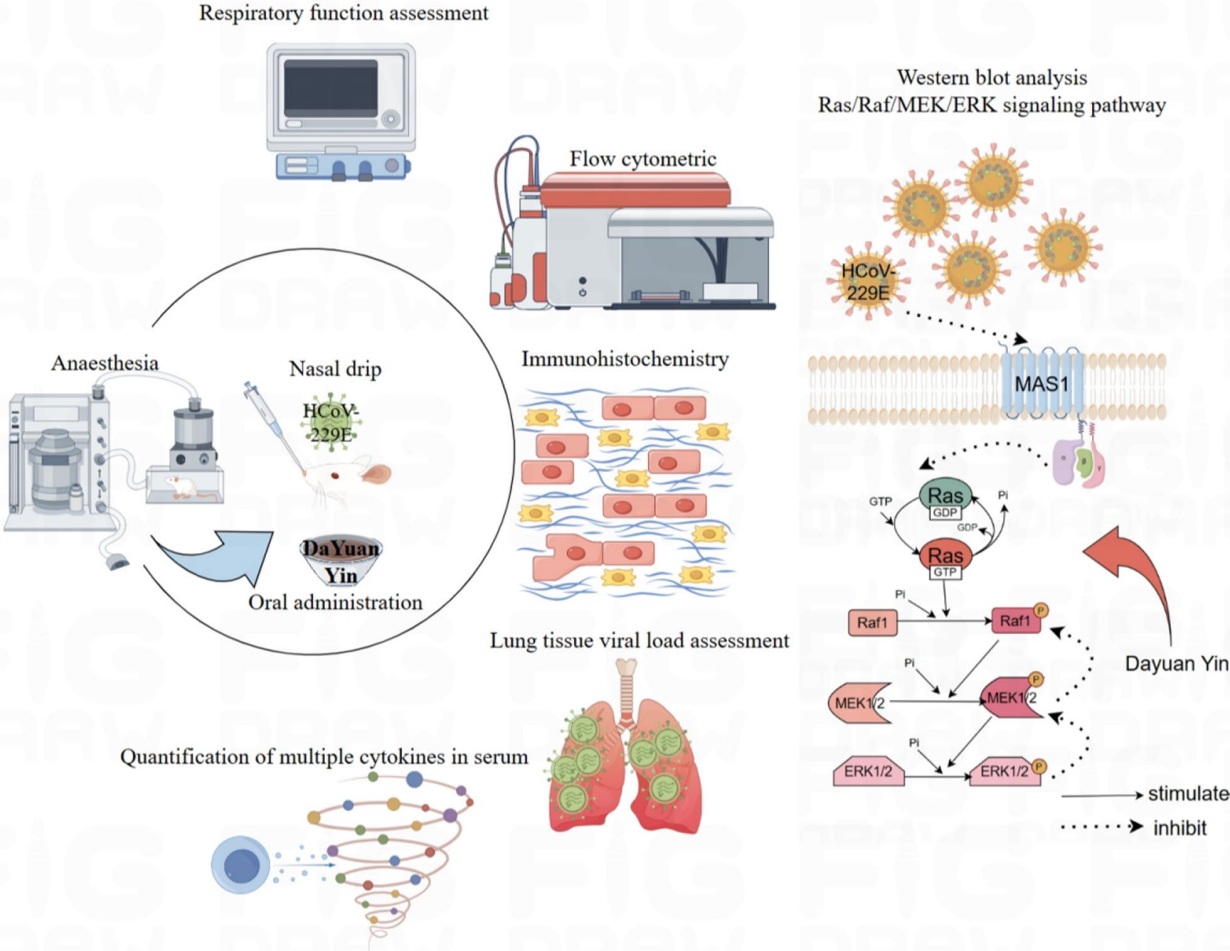

## Introduction

Viral pneumonia arises from respiratory viral infections that begin in the nasal cavity and pharynx, then progress downward, leading to inflammation of the lungs. Typical symptoms include coughing, fever, and chest pain. Without appropriate treatment, it may lead to severe complications like sepsis, multiple organ failure, and acute respiratory distress syndrome, all of which can be fatal [1]. Viral pneumonia is capable of causing large-scale outbreaks, exemplified by the COVID-19 pandemic that emerged at the end of 2019, thereby constituting a significant public health threat [2, 3]. Notable causative pathogens include influenza virus, herpes virus, respiratory syncytial virus (RSV), and coronaviruses such as Human Coronavirus 229E (HCoV-229E), which is prevalent among the population and particularly affects children in China [4, 5]. While infections from common coronaviruses are typically mild and self-limiting, they can lead to severe or fatal outcomes in vulnerable populations such as children, the elderly, and immunocompromised individuals [6].

The primary treatments for viral pneumonia currently include antiviral medications and corticosteroids. Antivirals like neuraminidase inhibitors, hemagglutinin inhibitors, M2 ion channel blockers, and RNA polymerase inhibitors have demonstrated efficacy in managing influenza virus pneumonia, though their effectiveness against other viral pneumonias lacks substantial clinical data [7–9]. Corticosteroid therapy, while commonly used to reduce inflammatory responses, remains controversial due to its pathogen-dependent effectiveness, and associated adverse reactions [10, 11]. Moreover, the rapid mutation rates of viruses contribute to the uncertainty of these treatments' long-term efficacy. Alternative treatments such as immunoglobulins, convalescent plasma, and respiratory support are available, yet they do not prevent viral transmission and have been linked to high failure rates and potential harm [12, 13]. Despite advances in addressing viral respiratory infections, the development of treatments that mitigate inflammatory response induced by pathogens with minimal side effects is still urgently needed.

Traditional Chinese Medicine (TCM) offers promising alternatives for the treatment of viral pneumonia. Dayuan Yin (DYY), a classic formula from Wu Youxing's "Treatise on Pestilence" during the Ming Dynasty, comprises seven herbs: *Areca catechu*, *Magnolia officinalis*, *Anemarrhena asphodeloides*, *Paeonia lactiflora*, *Scutellaria baicalensis*, *Glycyrrhiza glabra*, and *Amomum tsao-ko*. It has been utilized historically in the treatment of lung diseases and in managing epidemics. Clinically, DYY and its modifications are applied across a variety of systems to treat conditions ranging from fevers and skin diseases to more complex disorders like influenza, atypical pneumonia, AIDS, and complications from COVID-19, where it has been noted to improve symptoms, reduce disease duration, and enhance prognosis [14–19]. The pharmacological actions of DYY by which it acts in viral pneumonia still warrant further exploration. This study aimed to investigate these mechanisms and the therapeutic effects of DYY on a viral pneumonia model established through nasal inoculation with HCoV-229E virus.

## Results

### Dayuan Yin improved respiratory function in HCoV-229E infected mice

**Fig. 1:**
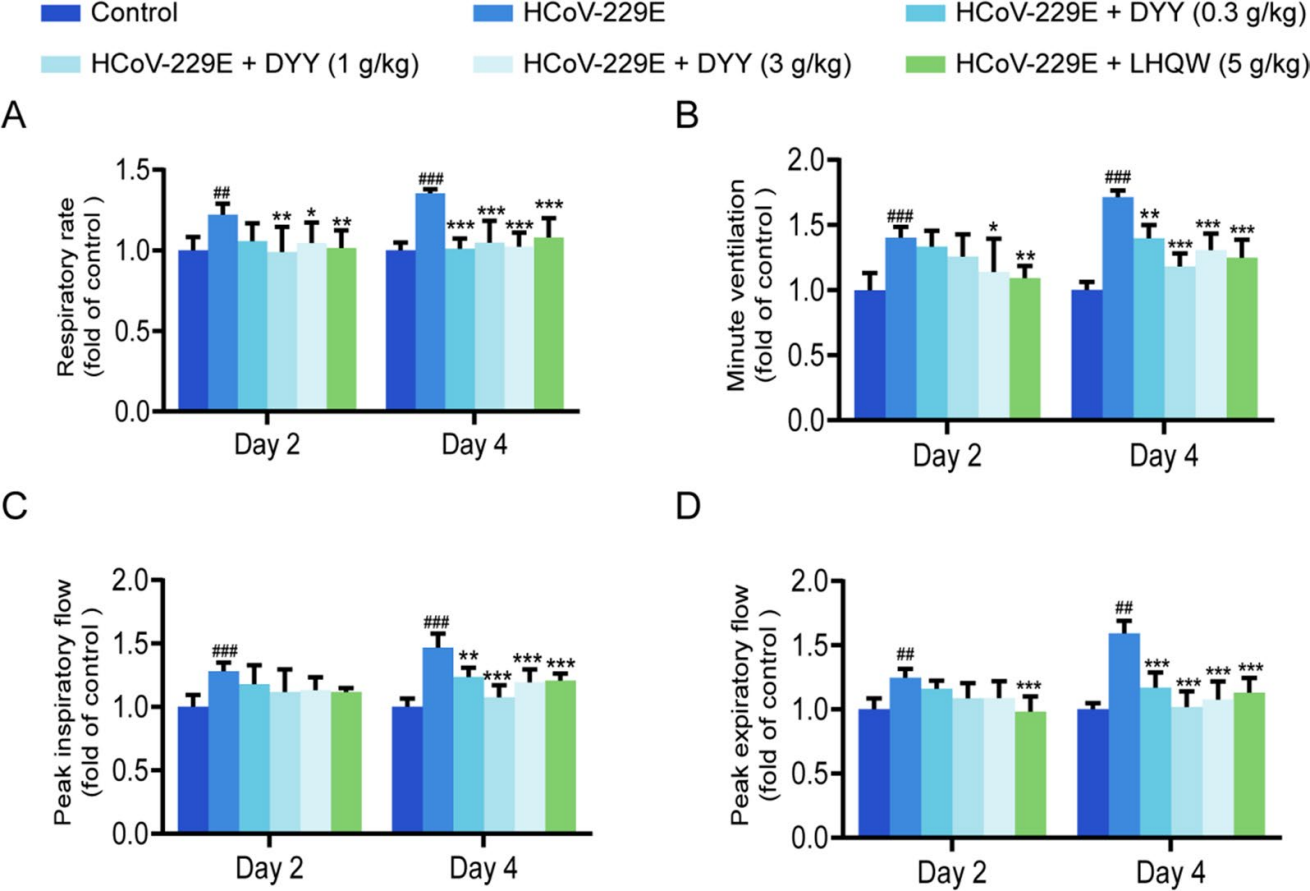
Dayuan Yin improved respiratory function in HCoV-229E infected mice. **A** Effects of DYY on respiratory rate; **B** Effects of DYY on minute ventilation; **C** Effects of DYY on peak inspiratory flow; **D** Effects of DYY on peak expiratory flow. n = 6. The results were expressed as mean ± SD. Statistical significance was denoted as follows: ^##^*P* < 0.01, ^###^*P* < 0.001 compared to the normal control group; ^*^P < 0.05, ^**^P < 0.01, ^***^P < 0.001 compared to the model group

Compared to the control group, the experimental mice showed significant increases in respiratory rate (F) on days two and four after modeling (^##^*P* < 0.01, ^*###*^*P* < 0.001, Fig. 1A). Following administration of 0.3 g/kg DYY, there was a non-significant reduction in F on day two, but a significant reduction by day four (^***^P < 0.001). With 1 g/kg DYY, F significantly decreased on both days (^**^P < 0.01, ^***^P < 0.001). Similarly, with 3 g/kg DYY, F significantly decreased on both days (^*^P < 0.05, ^***^P < 0.001). Treatment with 5 g/kg of LHQW resulted in a non-significant reduction in F on day two, but a significant reduction by day four (^***^P < 0.001).

Minute ventilation (MV) was significantly increased in the experimental group on both days (^###^*P* < 0.001, ^###^*P* < 0.001, Fig. 1B). It showed no significant reduction on day two after administration of 0.3 g/kg DYY but was significantly reduced by day four (^**^P < 0.01). With 1 g/kg DYY, MV significantly decreased by day four (^***^P < 0.001), and with 3 g/kg DYY, it decreased significantly on both days (^*^P < 0.05, ^***^P < 0.001). Treatment with 5 g/kg LHQW led to significant decreases on days two and four (^**^P < 0.01, ^***^P < 0.001).

Peak inspiratory flow (PIF) was significantly increased in the experimental group on both days (^###^*P* < 0.001, ^###^*P* < 0.001, Fig. 1C). It showed no significant reduction on day two after treatment with 0.3 g/kg DYY but was significantly reduced by day four (^**^P < 0.01). The same trend was observed with 1 g/kg and 3 g/kg DYY, with significant decreases by day four (^***^P < 0.001, ^***^P < 0.001). Treatment with 5 g/kg LHQW resulted in no significant change on day two but a significant decrease by day four (^***^P < 0.001).

Peak expiratory flow (PEF) was significantly higher in the experimental group on both days (^##^*P* < 0.01, ^##^*P* < 0.01, Fig. 1D). With 0.3, 1 and 3 g/kg DYY, PEF showed no significant change on day two but decreased significantly by day four (^***^P < 0.001 for all). Treatment with 5 g/kg LHQW led to no significant reduction on day two but a significant decrease by day four (^***^P < 0.001).

### Dayuan Yin regulated serum cytokine levels in HCoV-229E infected mice

**Fig. 2:**
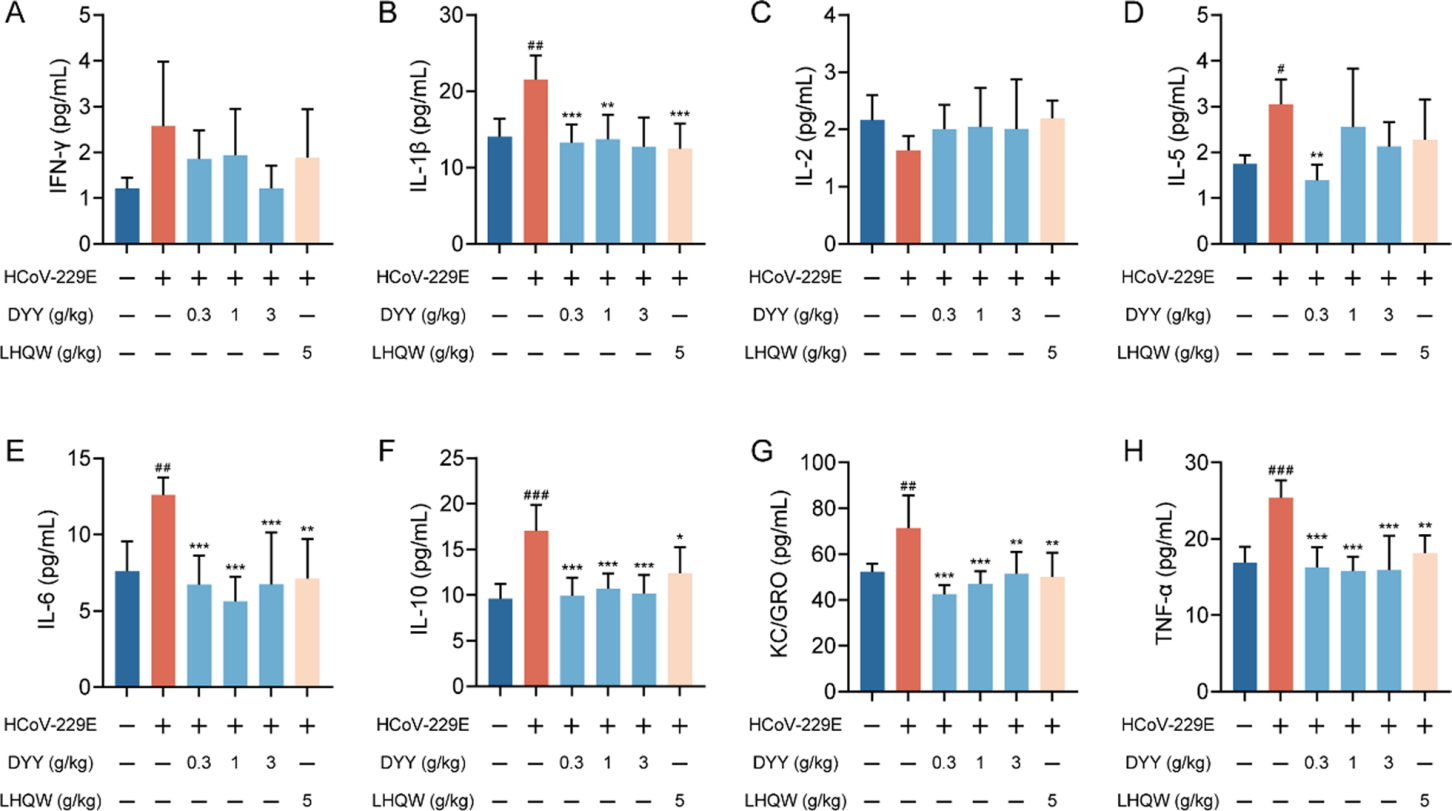
Dayuan Yin reduced cytokine levels in the serum of HCoV-229E infected mice. **A** Serum levels of IFN-γ; **B** Serum levels of IL-1β; **C** Serum levels of IL-2; **D** Serum levels of IL-5; **E** Serum levels of IL-6; **F** Serum levels of IL-10; **G** Serum levels of KC/GRO; **H** Serum levels of TNF-α. n = 6. The results are expressed as mean ± SD. Statistical significance was denoted as follows: ^#^*P* < 0.05, ^##^*P* < 0.01, ^###^*P* < 0.001 compared to the normal control group; ^*^P < 0.05, ^**^P < 0.01, ^***^P < 0.001 compared to the model group

Using multiplex immunoassays based on electrochemiluminescence to measure the levels of inflammatory cytokines in serum, 8 out of 10 cytokines were successfully detected. The contents of IL-4 and IL-12p70 were below the detection limit and thus not detected. There were no significant differences in the levels of IFN-γ and IL-2 either between the model and the normal control group or between the treated and model groups (Fig. 2A and C).

There was a significant increase in IL-1β levels in the model group compared to the normal control group (^##^*P* < 0.01, Fig. 2B). Both DYY (0.3 and 1 g/kg) and LHQW treatments significantly reduced IL-1β levels (^***^P < 0.001, ^**^P < 0.01, ^***^P < 0.001). IL-5 showed a significant increase in the model group compared to the normal control group (^#^*P* < 0.05, Fig. 2D). Treatment with 0.3 g/kg DYY significantly reduced IL-5 levels (^**^P < 0.01), but higher doses of DYY and LHQW did not show a significant difference. IL-6 levels were significantly higher in the model group compared to the normal control group (^##^*P* < 0.01, Fig. 2E). Both DYY (0.3, 1 and 3 g/kg) and LHQW treatments significantly reduced IL-6 levels (^***^P < 0.001, ^***^P < 0.001, ^***^P < 0.001, ^**^P < 0.01). IL-10 levels were markedly increased in the model group compared to the normal control group (^###^*P* < 0.001, Fig. 2F). Both DYY (0.3, 1 and 3 g/kg) and LHQW treatments significantly reduced IL-10 levels (^***^P < 0.001, ^***^P < 0.001, ^***^P < 0.001, ^*^P < 0.05). KC/GRO also showed a significant increase in the model group compared to the normal control group (^##^*P* < 0.01, Fig. 2G). Treatment with DYY (0.3, 1 and 3 g/kg) and LHQW significantly decreased KC/GRO levels (^***^P < 0.001, ^***^P < 0.001, ^**^P < 0.01, ^**^P < 0.01). TNF-α levels showed a highly significant increase in the model group compared to the normal control group (^###^*P* < 0.001, Fig. 2H). Both DYY (0.3, 1 and 3 g/kg) and LHQW treatments significantly reduced TNF-α levels (^***^P < 0.001, ^***^P < 0.001, ^***^P < 0.001, ^**^P < 0.01). These results highlighted the significant changes in specific inflammatory cytokines due to HCoV-229E infection and the effective modulation of these cytokines by treatment with DYY = , demonstrating its potential as therapeutic agent in reducing inflammation.

### Dayuan Yin alleviated lung and tracheal damage in HCoV-229E infected mice

**Fig. 3:**
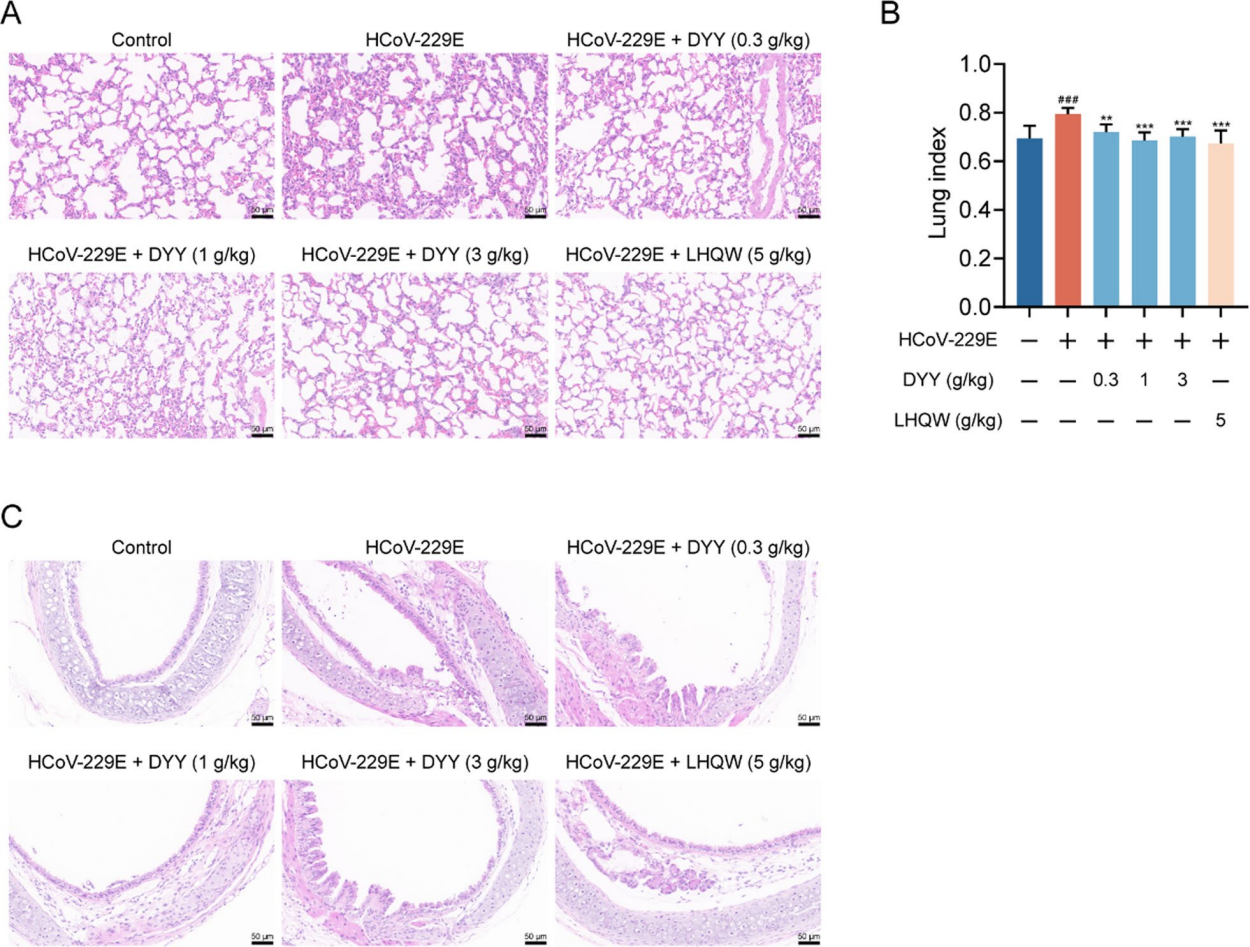
Dayuan Yin alleviated lung and tracheal damage in mice infected with HCoV-229E and reduced lung index. **A** Pathological changes in lung tissue; **B** Lung index (n = 8); **C** Pathological changes in tracheal tissue. The results were expressed as mean ± SD. Statistical significance was denoted as follows: ^#^*P* < 0.05, and ^###^*P* < 0.001 compared to the normal control group; ^***^P < 0.001 compared to the model group

Immunohistochemical analyses revealed that, following HCoV-229E infection in mice, the model control group exhibited widened and thickened alveolar interstitium, pulmonary edema, and destruction of alveolar structures. Treatment with DYY significantly ameliorated these pathologies, with LHQW also showing improvement effects (Fig. 3A). Compared to the normal group, the lung index in the model group significantly increased (^###^*P* < 0.001). Administration of DYY at 0.3, 1, and 3 g/kg, as well as the positive control LHQW, significantly reduced the lung index (^*^P < 0.01, ^***^P < 0.001, ^***^P < 0.001, ^***^P < 0.001, respectively; Fig. 3B). Tracheal cellular structures were also compromised, with evidence of inflammatory cell infiltration. Treatment with DYY significantly alleviated these conditions, with more pronounced effects observed at medium and high doses of DYY. And the positive control, LHQW, also demonstrated therapeutic effects on these damages (Fig. 3C). These results demonstrated that DYY significantly mitigated pulmonary and tracheal damage caused by HCoV-229E infection in mice, highlighting their potential therapeutic value in managing viral respiratory infections.

### Dayuan Yin increased CD4/CD8 ratio in splenic cells of HCoV-229E infected mice

**Fig. 4:**
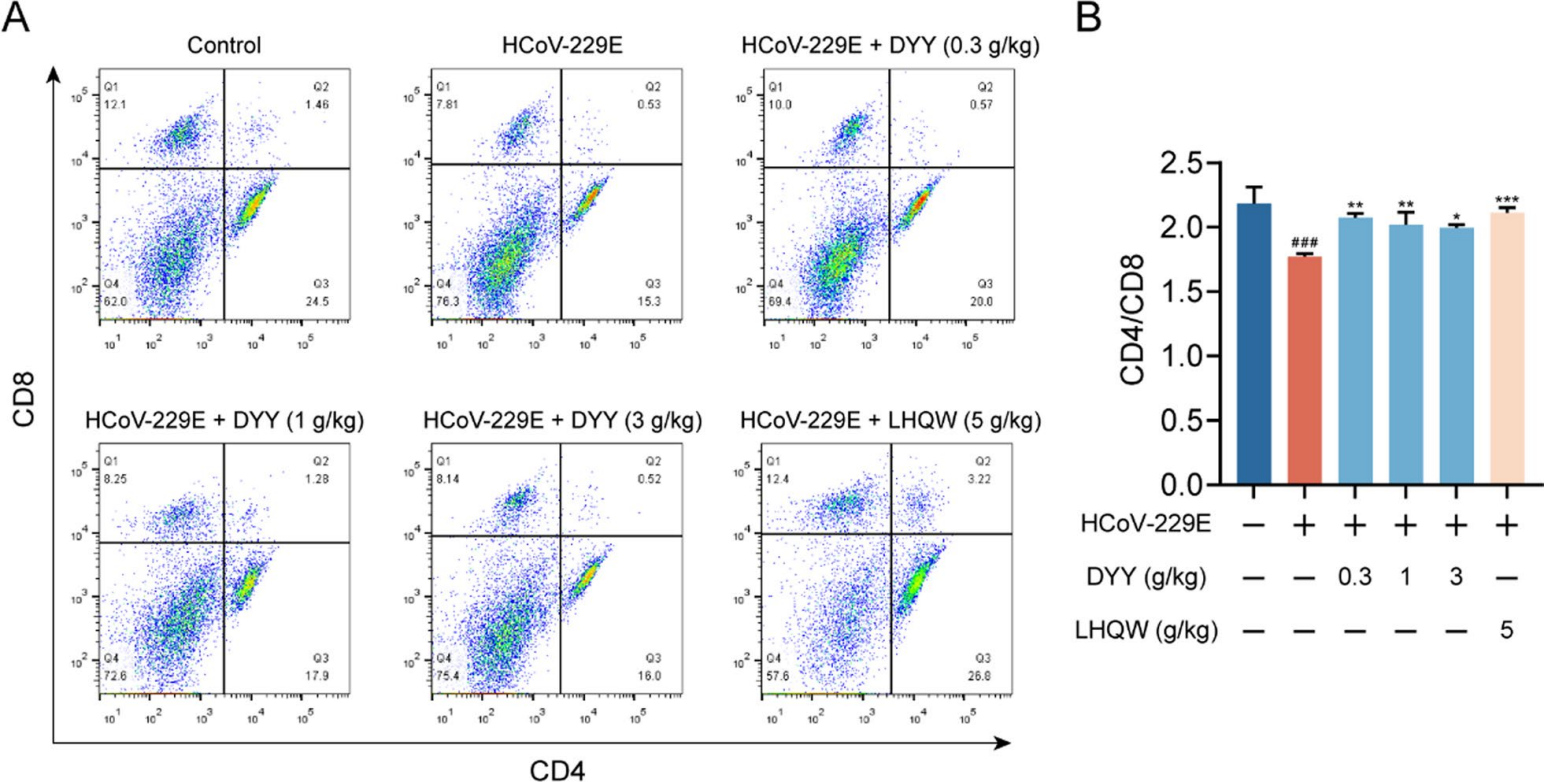
Dayuan Yin elevated the CD4/CD8 ratio in splenic cells of HCoV-229E infected mice. **A** Flow cytometry plots for CD4 and CD8; **B** Ratio of CD4 to CD8 (n = 3). The results were expressed as mean ± SD. Statistical significance is denoted as follows: ^#^*P* < 0.05, and ^###^*P* < 0.001 compared to the normal control group; ^*^P < 0.05, ^**^P < 0.01, and ^***^P < 0.001 compared to the model control group

Flow cytometric analysis of splenic cells revealed a significant decrease in the CD4/CD8 ratio in the model group compared to the normal control group (^###^*P* < 0.001). Treatment with DYY at doses of 0.3, 1, and 3 g/kg and LHQW significantly enhanced the CD4/CD8 ratio (^**^P < 0.01, ^**^P < 0.01, ^*^P < 0.05, ^***^P < 0.001, respectively, Fig. 4A and B). The study highlighted the immunomodulatory effects of DYY and LHQW in managing immune cell dysregulation in this model.

### Dayuan Yin reduced viral load in lung tissues of HCoV-229E infected mice

**Fig. 5:**
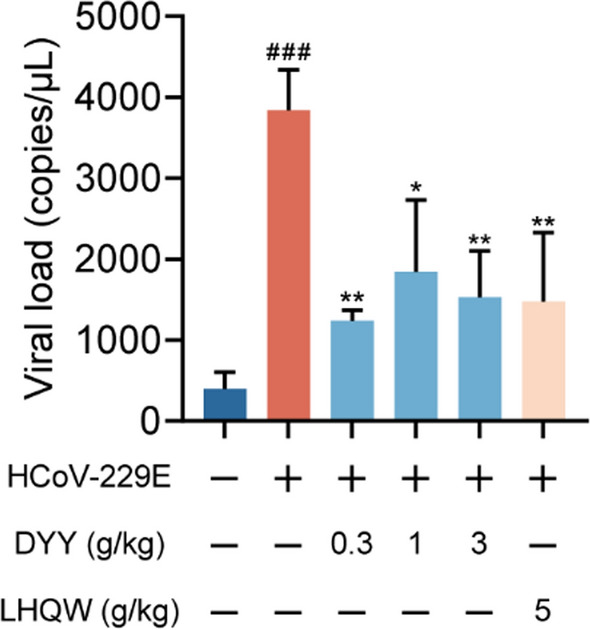
Dayuan Yin reduced viral load in lung tissue of mice infected with HCoV-229E. n = 3. The results were expressed as mean ± SD. Statistical significance is denoted as follows: ^###^*P* < 0.001 compared to the normal control group; ^*^P < 0.05, and ^**^P < 0.01 compared to the model control group

In the present study, HCoV-229E infected mice exhibited a significant increase in viral load within the lung tissues as compared to uninfected normal mice, indicating a successful establishment of the infection model (^###^*P* < 0.001). Subsequent treatment with DYY at dosages of 0.3, 1, and 3 g/kg, as well as with the positive control LHQW, was evaluated for its antiviral efficacy. Results demonstrated a significant reduction in viral concentrations in the lung tissues following treatment. Specifically, the 0.3, 1 and 3 g/kg doses of DYY and LHQW all showed significant decreases in viral load (^**^P < 0.01, ^*^P < 0.05, ^**^P < 0.01, ^**^P < 0.01). This reduction underscored the potential of DYY as effective antiviral treatments against HCoV-229E (Fig. 5).

### Dayuan Yin increased the expression of MAS1 and inhibited the activation of Ras/Raf1/MEK/ERK signaling pathway

**Fig. 6:**
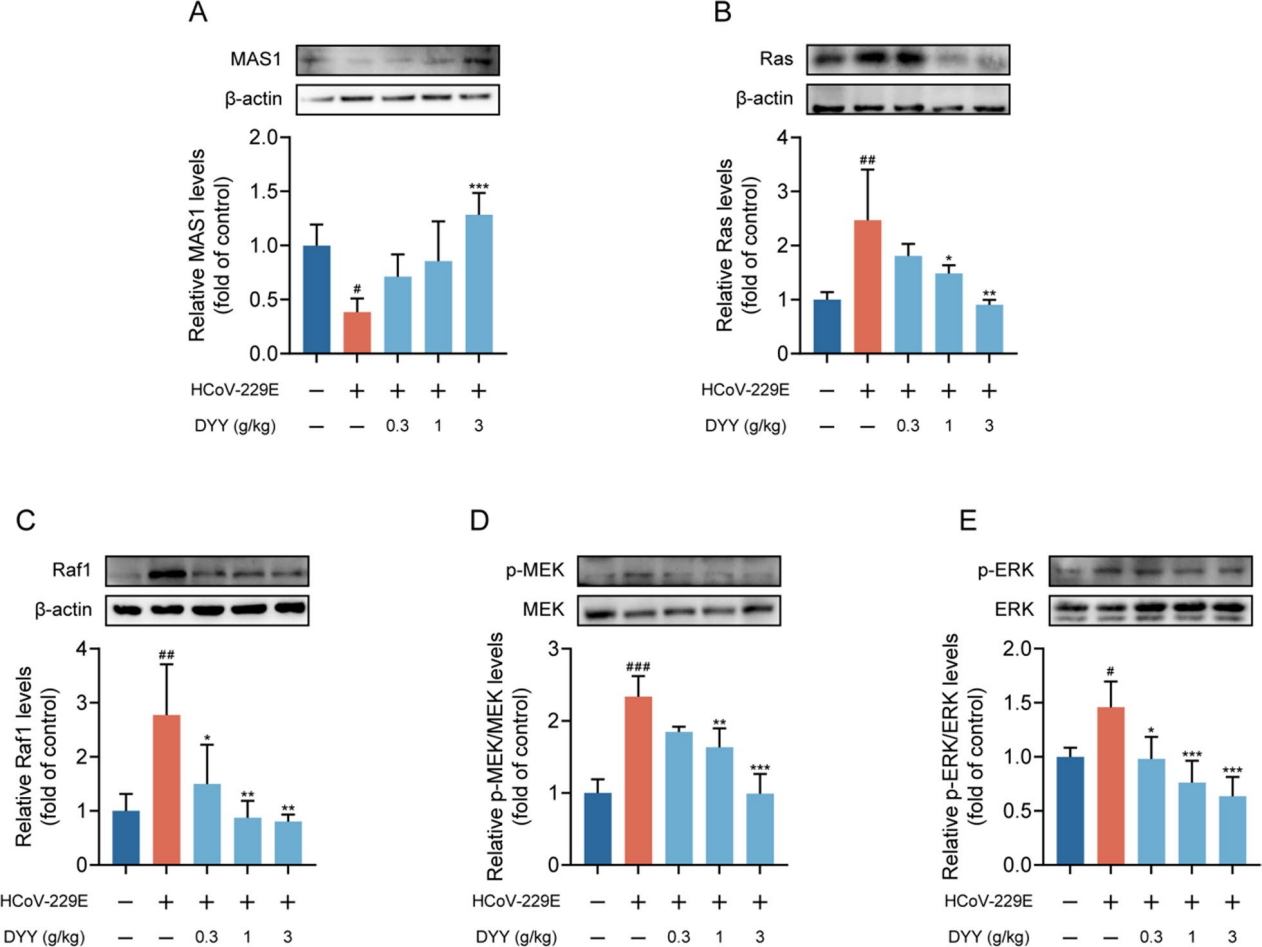
Dayuan Yin promoted the expression of MAS1 and inhibited the activation of the Ras/Raf1/MEK/ERK signaling pathway. **A** Levels of MAS1 protein; **B** Levels of Ras protein; **C** Levels of Raf1 protein; **D** Levels of p-MEK1/2/MEK1/2; **E** Levels of p-ERK1/2/ERK1/2 proteins. n = 4. The results were expressed as mean ± SD. Statistical significance is denoted as follows: ^#^*P* < 0.05, ^##^*P* < 0.01, and ^###^*P* < 0.001 compared to the normal control group; ^*^P < 0.05, ^**^P < 0.01, and ^***^P < 0.001 compared to the model group

Western blot analysis revealed that in the HCoV-229E infected model group, levels of MAS1 protein were significantly decreased compared to the normal control group (^#^*P* < 0.05). Treatment with DYY at a dosage of 3 g/kg significantly increased the levels of MAS1 protein (^***^P < 0.001, Fig. 6A). For Ras protein, there was a significant increase in levels in the HCoV-229E infected model group compared to the normal control group (^##^*P* < 0.01). Treatment with DYY at dosages of 1 and 3 g/kg significantly reduced the levels of Ras protein (^*^P < 0.05, ^**^P < 0.01, Fig. 6B). Regarding Raf1 protein, there was a significant increase in levels in the HCoV-229E infected model group compared to the normal control group (^##^*P* < 0.01). Treatment with DYY at dosages of 0.3, 1, and 3 g/kg significantly reduced the levels of Raf1 protein (^*^P < 0.05, ^**^P < 0.01, ^**^P < 0.01, Fig. 6C). For p-MEK/MEK levels, there was a significant increase in the HCoV-229E infected model group compared to the normal control group (^###^*P* < 0.001). Treatment with DYY at dosages of 1 and 3 g/kg significantly reduced the levels of phosphorylated MEK1/2 (^**^P < 0.01, ^***^P < 0.001, Fig. 6D). For p-ERK/ERK levels, there was a significant increase in the model group compared to the normal control group (^#^*P* < 0.05). Treatment with DYY at dosages of 0.3, 1, and 3 g/kg significantly reduced the levels of phosphorylated ERK1/2 (^*^P < 0.05, ^***^P < 0.001, ^***^P < 0.001, Fig. 6E). These results indicated that DYY effectively modulated key signaling pathways involved in the response to HCoV-229E infection, offering potential mechanisms through which DYY might exert its therapeutic effects.

## Discussion

Viral pneumonia, a complex multi-system disorder characterized by intense inflammation and systemic symptoms, challenges both innate and adaptive immune responses [20]. This study employed HCoV-229E to infect male BALB/c mice, thereby establishing a viral pneumonia model. In fact, mice are naturally not susceptible to HCoV-229E primarily because they lack the receptor necessary for viral entry. However, this resistance does not mean that infection is completely unachievable. By selecting specific strains and employing certain methods of administration, it is still possible to infect mice with the virus. According to a previous study, through the screening of infection system, strain and frequency of infection, it was found that the model of young ordinary BALB/c mice was successfully constructed via two nasal administrations. The dosing group started dosing on the first day of model construction for a total of five days, on the fifth day, the model was successfully established, and the changes of lung and systemic indicators were clinically consistent [21]. The receptor of HCoV-229E is aminopeptidase N (APN) [22]. Another study utilized an adenovirus vector expressing the human aminopeptidase N (hAPN), to create susceptible mouse model. This approach allowed for the rapid construction of a mouse model sensitive to HCoV-229E infection. Following infection, the mice developed interstitial pneumonia. The virus could sustain replication for approximately 7 days, making this model useful for evaluating antiviral drugs and vaccines [23]. Therefore, compared to the hAPN transgenic model, the ordinary mouse model used in this study clearly has certain limitations, primarily in terms of infection efficiency and severity. Despite these shortcomings, the ordinary model remains effective for evaluating the efficacy of drugs.

Most patients with viral pneumonia have a rapid onset, often accompanied by respiratory symptoms such as cough, white mucus sputum, sore throat and increased respiratory rate. PIF and PEF are indicators of lung function and refer to the fastest flow rate during inhalation/exhalation using maximum force and speed, which can better reflect the patency of the airway. MV refers to the total amount of gas entering or exiting the lung per minute, which is the product of tidal volume and F, it is used to measure the elasticity of thoracic lung tissue, airway resistance and respiratory muscle strength [24, 25]. DYY treatment has demonstrated significant improvements in respiratory function in animal models infected with HCoV-229E, highlighting its potential in managing acute respiratory symptoms and systemic effects of viral infections. This study confirmed that DYY administration notably reduced the HCoV-229E infected mice’s respiratory rate, PIF, PEF, and MV, indicating improved lung function and airway patency.

In the immune response process of the body, cells interact and restrict each other by secreting cytokines, forming a complex and orderly cytokine regulatory network to maintain the equilibrium and homeostasis of the immune system [26]. However, under some external stimuli, such as severe infection, the excessive inflammatory response breaks this balance, and the body overproduces a variety of cytokines in a short time. According to the structure and function of different cytokines, there are mainly six categories, including interferon, interleukin, Chemokine, colony stimulating factor, tumor necrosis factor, and growth factor. In the face of viral infection, an excessive inflammatory response can lead to a cytokine storm, causing significant tissue damage and systemic effects [27]. The anti-inflammatory effects of DYY were evident through its regulation of cytokine production. Our findings suggested that DYY could substantially modulate the cytokine environment, reducing serum levels of key pro-inflammatory cytokines like IL-1β, IL-6, TNF-α, and others, thereby mitigating inflammation.

The ratio of CD4/CD8 is an important indicator of immune regulation, and its decrease indicates immune dysfunction, which is common in viral infection [26, 28, 29]. The reparative properties of DYY are critical in the context of viral damage to lung and tracheal tissues, commonly seen in severe viral pneumonia cases that progress to acute respiratory distress syndrome. This study observed that DYY treatment effectively lowered the lung index, reduced edema, and repaired cellular structures. Furthermore, DYY significantly adjusted the CD4/CD8 ratio, which are vital for an effective immune response to viral infections.

Viruses are typically transmitted through droplets or direct inhalation, entering the respiratory tract where they bind to specific cellular receptors. Following adhesion, viruses release their nucleic acids into the host cells, leveraging the cellular machinery to produce more virions. These new virions are then transported to the cell surface and released, completing the cycle of viral proliferation. During this process, the host cells often die, resulting in damage to the lung epithelial tissue. Concurrently, the host immune system responds by releasing a substantial number of cytokines, chemical mediators, and enzymes to combat the virus infection, potentially leading to a cytokine storm and oxidative stress [30]. DYY has shown potent antiviral effects by reducing the viral load in lung tissues, suggesting an inhibition of viral replication. Moreover, the modulation of immune response by DYY, particularly its effects on lymphocyte dynamics and cytokine profiles, underscores its role in not only combating the virus but also in regulating the body's defense mechanisms against overreaction to virus.

The Ras/Raf1/MEK/ERK signaling pathway is a critical cascade of proteins that transmits signals from the cell surface to the DNA in the nucleus, thereby influencing gene expression and affecting cellular growth, differentiation, and survival. In the context of viral infections, including HCoV-229E, the modulation of this pathway can be pivotal. ERK1/2 signaling pathway can inhibit Th1 cell differentiation, thereby reducing and inducing Th2 cell differentiation and regulating cytokine and inflammatory response [31]. ERK has a regulatory effect on cell proliferation, differentiation, cycle and other processes. When stimulated by oxidative stress, ERK is regulated by upstream signals and transferred to the nucleus through phosphorylation modification to activate the downstream pathway. MEK, as an upstream regulator of ERK, can regulate the phosphorylation and activation of ERK, thus affecting cell functions [32, 33]. Ras, as a small G protein, converts between binding GTP and GDP, binding GTP plays a role in activating Raf, binding GDP signal is turned off, and its activation is regulated by Grb2-Sos, ERK signaling pathway from extracellular cytokines, receptors, Grb2-Sos complexes, Ras, RAF, and RB2-SOS. Mutations or disorders may occur from Raf to MEK1/2, and their regulation at all levels can become targets for disease treatment. Ras/ERK signaling pathway belongs to one of the three branches of the MAPK cascade signaling pathway. Classical activation is initiated by ligands binding to receptor tyrosine kinases on the cell surface, followed by Raspberry, Raf, MEK, ERK, which can regulate cell proliferation, survival, differentiation, angiogenesis, migration and other cell functions [34]. Viruses often manipulate host cellular signaling pathways to create an environment conducive to their replication and spread. The activation of this pathway during viral infections could lead to altered immune responses and cellular apoptosis, which can impact the pathogenesis and outcomes of the infection.

In the research presented, the western blot analysis indicated the specific alterations in the components of the Ras/Raf1/MEK/ERK pathway following HCoV-229E infection. It is observed that the infection led to a significant increase in Ras protein levels, indicating an activation of this pathway. Subsequent upregulation of downstream proteins such as Raf1, phosphorylated MEK1/2, and phosphorylated ERK1/2 further corroborated the activation of this signaling cascade. These changes suggested that HCoV-229E might exploit this pathway to promote viral replication or to evade host immune responses. Moreover, the study revealed the therapeutic potential of DYY in modulating this activated signaling pathway during HCoV-229E infection. DYY treatment significantly reduced the levels of Ras, Raf1, phosphorylated MEK1/2, and phosphorylated ERK1/2 proteins. Notably, the reduction in these proteins correlated with increased dosage of DYY, emphasizing its dose-dependent efficacy in inhibiting the activation of this pathway. This inhibition likely contributed to a controlled cellular environment, potentially limiting viral replication and mitigating virus-induced pathogenic effects.

**Fig. 7:**
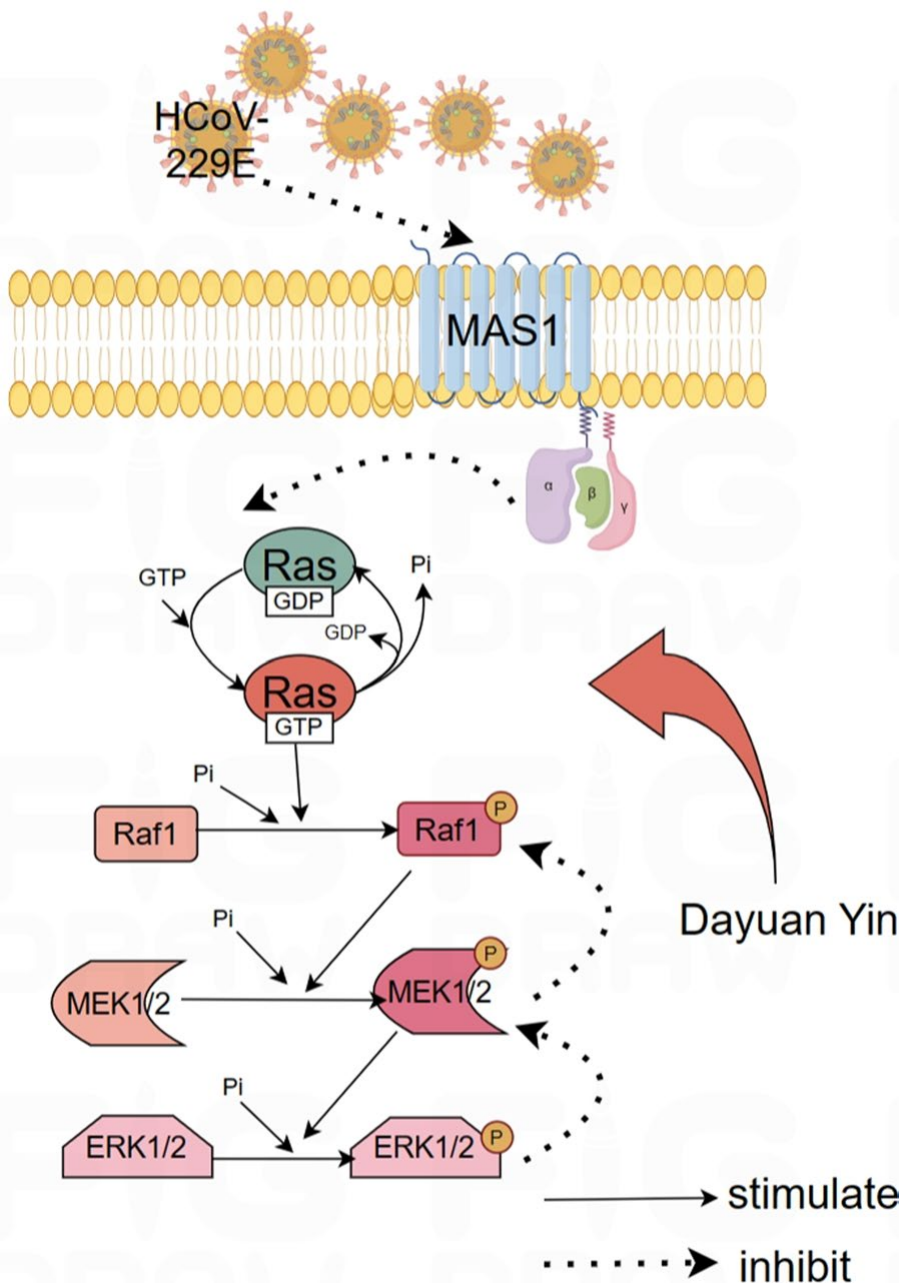
Mechanism diagram of Dayuan Yin's regulation of the Ras/Raf/MEK/ERK signaling pathway

Additionally, the observed increase in MAS1 protein levels upon treatment with DYY at higher doses further supported the therapeutic effects of DYY. The MAS1 receptor, part of the renin-angiotensin system, has implications in inflammation and cellular stress responses, which are often manipulated by viral infections. Mas gene is highly conserved, and the protein it encodes is a typical G-protein-coupled receptor. Drug regulation of Mas receptor can play an important role in regulating neuroplasticity, memory and anxiety, including anti-angiogenesis, vasodilation, anti-proliferation, anti-fibrosis and anti-thrombosis [35]. By increasing MAS1 protein levels, DYY might help restore normal cellular function and enhance antiviral responses (Fig. 7).

## Conclusion

In summary, DYY treatment improved the respiratory function of mice infected with the HCoV-229E virus, exerted anti-inflammatory, antiviral, and immunomodulatory effects, and repair lung tissue damage. The action mechanism might be related to the increased expression of MAS1 and the inhibition of the Ras/Raf/MEK/ERK signaling pathway.

## Materials and methods

### Drugs and reagents

HCoV-229E (VR-740, American Type Culture Collection, Pfu ≥ 5 × 10^3^ per/mL. Isoflurane (R510-22-10, RWD Life science Co.,LTD), TRIzol^®^ Reagent (ET101-01-V2, TransGen Biotech Co., LTD), Paraformaldehyde (P1110, Beijing Solarbio Life Sciences Co., LTD), DNase/RNase-Free Water (R1600, Beijing Solarbio Life Sciences Co., LTD), Roswell Park Memorial Institute 1640 (31800, Beijing Solarbio Life Sciences Co., LTD). PerCP/Cyanine5.5 anti-mouse CD4 Antibody (100434, BioLegend, Inc.), PE/Cyanine7 anti-mouse CD8a Antibody(100722, BioLegend, Inc.)

For the preparation of DYY Extract, the following herbs were used: *Areca catechu* (300 g), *Magnolia officinalis* (150 g), *Anemarrhena asphodeloides* (150 g), *Paeonia lactiflora* (150 g), *Scutellaria baicalensis* (150 g), *Glycyrrhiza glabra* (75 g), and *Amomum tsao-ko* (75 g). Each herb was weighed and mixed uniformly. The mixture was placed in a 1000 mL round-bottom flask, and water was added at 5, 4, and 3 times the volume of the herbs respectively for each sequential extraction. Each extraction was conducted by reflux heating for 1.5 h. The combined filtrates were then concentrated under reduced pressure to achieve a yield of 25.5%, resulting in a crude extract concentration of 1.85 g/mL.

Lianhua Qingwen granule (LHQW, batch number: Z20100040) was purchased from Beijing Yiling Pharmaceutical Co., Ltd. 2.5g LHQW granule was accurately weighed and mixed with pure water preheated at 70 ℃ to fully dissolve it, so as to obtain 5 g/kg LHQW solution.

### Establishment of the HCoV-229E mouse model and grouping

Male BALB/c mice, weighing 12-14 g, were purchased from Beijing HFK Bioscience Co., Ltd, certificate number SCXK2019-008. Mice were kept at 22 ± 2°C, with free access to food and water, under a half-day light and dark cycle. Mice were randomly divided into six groups: normal control group, HCoV-229E infection group, HCoV-229E + DYY group (0.3, 1, 3 g/kg), and a positive control LHQW (5 g/kg) group. The animal study protocol was approved by the Scientific Ethics Review Board of Qingdao Marine Biomedical Research Institute (Permit NO. E-MBWDYY-2023–24). Both cells and animals are operated in the ASBL-2 Biosafety Laboratory.

**Fig. 8:**
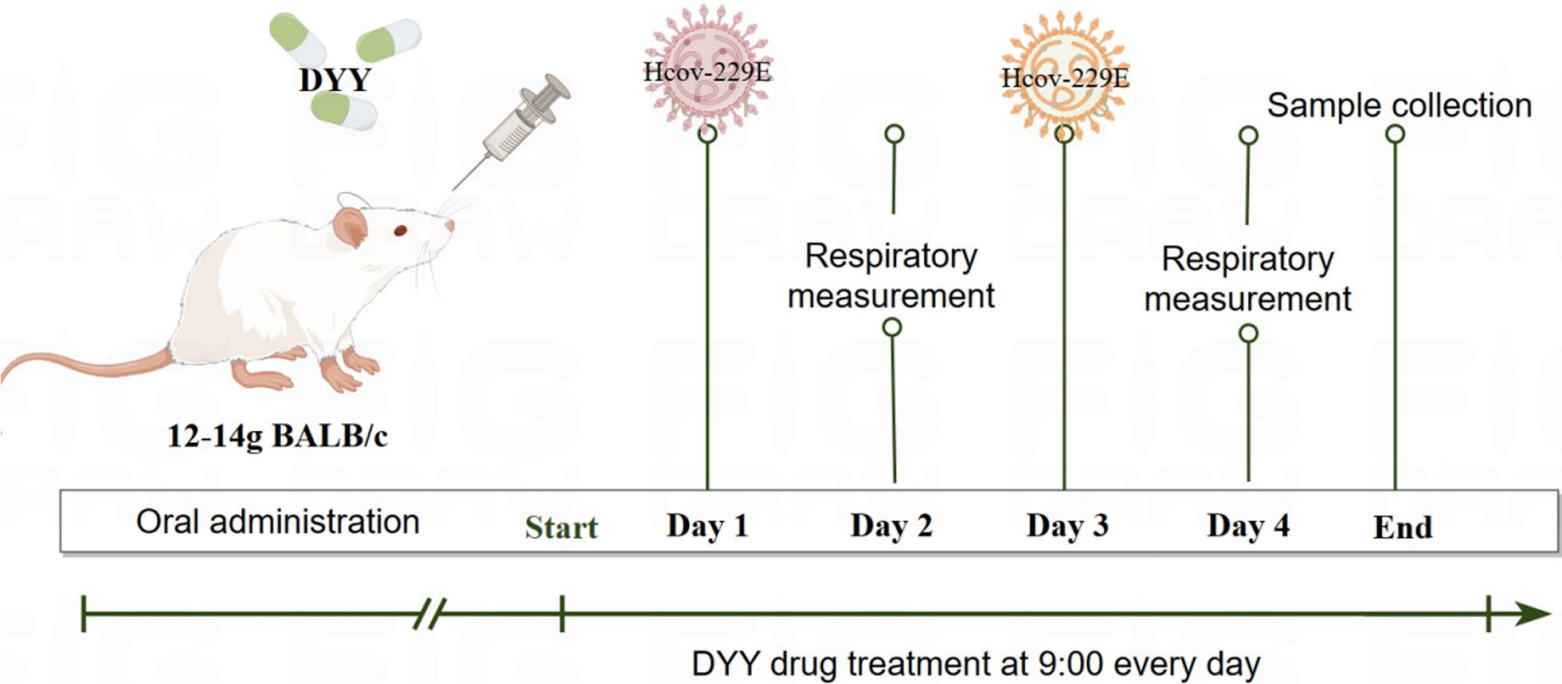
The experiment protocol of HCoV-229E infected pneumonia mice and treatment with DYY

Except for the control group, mice in all other groups were lightly anesthetized with isoflurane and subsequently infected intranasally with 1 × 10^6^ PFU/mL of HCoV-229E. Each mouse received 50 µL of the virus solution, with administrations given every other day for a total of two doses. Control mice were given the same volume of DMEM intranasal infusion. On the day of the first infection, the LHQW positive control group was administered 5 g/kg/day by gavage, while the DYY low, medium, and high dose groups received doses of 0.3, 1, and 3 g/kg/day, respectively, also by gavage. The normal control group were administered 0.5% CMC-Na with the same volume, once daily for four days (Fig. 8).

### Respiratory function assessment

On the second and fourth days of the experiment, to minimize the potential impact of medication on respiratory function, the respiratory functions of mice in each experimental group were assessed one hour before medication administration using a whole-body plethysmograph system (Shanghai TOW Intelligent Technology Co., Ltd.). Prior to commencing the experiments, the equipment was zero-calibrated, and flow parameters were set to 0.3 L/min. A bypass was opened to ensure normal breathing for the mice, who were then placed in the chamber for a 15-min acclimation period to ensure calmness, followed by recording 15 min of respiratory waveforms.

### Sample collection and lung index determination

On the fifth day of the experiment, mice were weighed before blood collection. Mice were deeply anesthetized using 3% isoflurane, and then the eyeballs were removed to take blood. When the blood was exhausted, the mice were euthanized by neck removal. After blood collection, the trachea, lungs underwent saline rinsing to remove excess blood, followed by absorption of any remaining moisture with filter paper, and subsequent lung weighing. The lung index (%) was calculated as the organ weight (g) divided by the body weight (g) multiplied by 100%.

Blood samples were stored in 1.5 mL Eppendorf tubes at room temperature for 2 h to allow for serum separation. Subsequently, they were centrifuged at 4°C in a pre-cooled low-temperature centrifuge at 3000 rpm for 15 min. The resulting supernatant was carefully collected and stored at -80°C for further analysis. The trachea and part of the left lung were fixed in tissue fixative, fresh spleen tissue was processed for cell extraction, and the remaining lung tissue was stored at -80°C for future analysis.

### Lung tissue viral load assessment

The lung tissue samples stored at -80 °C were placed in tissue grinding tubes, and 1 mL of Trizol reagent was added for tissue homogenization using an electric homogenizer. The tubes were then left at room temperature for 20 min before being centrifuged at 4 °C and 12,000 rpm for 10 min. The supernatant was transferred to a new centrifuge tube, to which 0.2 mL of chloroform was added, followed by shaking for 15 s and incubation at room temperature for 3 min to allow phase separation. After centrifugation at 4°C and 12,000 rpm for 15 min, the supernatant was transferred to a new centrifuge tube and mixed with 0.5 mL of isopropanol, then incubated at room temperature for 30 min. This was followed by centrifugation at 4 °C and 12,000 rpm for 10 min. The supernatant was discarded, and the RNA precipitate was washed with 1 mL of 75% ethanol and centrifuged at 4 °C and 7500 rpm for 5 min. The supernatant was removed, and the RNA precipitate was briefly dried for 5-10 min. The precipitate was then dissolved in 20 µL of DEPC water and stored at -80 °C. Viral RNA was detected using the Human Coronavirus 229E (HCoV-229E) SYBR RT-PCR Kit (lot number: 300141tdz, TIANDZ Biotechnology Co., Ltd.) following the manufacturer's instructions.

### Flow cytometric analysis of splenic cells

The freshly rinsed spleen was ground in 1640 medium and filtered through a 70 µm cell strainer. The cell suspension was centrifuged at 4 °C and 3000 rpm for 10 min, and the supernatant was discarded. The cell pellet was resuspended in 1 mL of red blood cell lysis buffer at room temperature for 3 min, followed by another centrifugation at 4 °C and 3000 rpm for 10 min. The cells were then resuspended in 500 µL of PBS and washed twice more. Each tube was then added with PerCP/Cyanine5.5 anti-mouse CD4 antibody and PE/Cyanine7 anti-mouse CD8a antibody and incubated in the dark for 30 min. The cells were washed once with PBS and analyzed using BD FACSCanto II Flow Cytometer.

### Immunohistochemistry

After fixation, the lung tissues of the mice underwent infiltration and embedding in paraffin following a gradient alcohol dehydration and xylene clearance process. Subsequently, the embedded tissues were sectioned and affixed onto slides. Following this, the sections underwent staining with hematoxylin and eosin (H&E). Finally, the stained sections were meticulously examined under a microscope to observe any pathological morphological changes in the lung tissues.

### Quantification of multiple cytokines in serum

Levels of multiple cytokines in serum were quantified using a hypersensitive multiplex electrochemiluminescence analyzer (MESO QuickPlex SQ 120MM, Meso Scale Discovery) and the V-PLEX Proinflammatory Panel 1 Mouse Kit (K15048D, Meso Scale Discovery), which includes ten cytokines: IFN-γ, IL-1β, IL-2, IL-4, IL-5, IL-6, IL-10, IL-12p70, KC/GRO, and TNF-α. Initially, 25 µL of serum was diluted twofold with Diluent 41. Standards were prepared using a fourfold gradient dilution. Both samples and standards (50 µL each) were then incubated at room temperature for 2 h. Subsequently, the plate was washed three times with 150 µL of wash buffer per well. Next, 25 µL of a 50-fold diluted antibody solution prepared with Diluent 45 was added to each well and incubated at room temperature for an additional 2 h. After three more washes with 150 µL of wash buffer per well, each well was treated with 150 µL of Read Buffer T. The plate was read immediately after the final addition.

### Western blot analysis

Using a BCA protein assay kit (Applygen Technologies Inc, Beijing, China), total proteins were extracted from the lung tissues and quantified. The proteins were then mixed with SDS buffer and boiled for 10 min. SDS-PAGE electrophoresis was performed, followed by the transfer of proteins to a PVDF membrane. The membrane was blocked with 5% skim milk at room temperature for 2 h. The proteins were then incubated with various antibodies overnight at 4 °C. After incubation, secondary antibodies were added and reacted for 2 h. The expression of various proteins was observed using a high-sensitivity chemiluminescence detection kit. The expressions of MAS1 (Abcam, ab235914, 1:2000 dilution), Ras (Abcam, ab180772, 1:1000 dilution), Raf1 (Abcam, ab181115, 1:1000 dilution), MEK1/2 (proteintech, 11049-1-AP, 1:5000 dilution), p-MEK1/2 (Cell Signaling Technology, 9121S, 1:1000 dilution), ERK1/2 (proteintech, 11257-1-AP, 1:5000 dilution), and p-ERK1/2 (proteintech, 28733-1-AP, 1:4500 dilution) in the lung tissues were assessed.

### Statistical analysis

Measurement data were expressed as mean ± standard deviation. Data were analyzed using GraphPad Prism software version 7.05. Comparisons were made using one-way ANOVA or two-way ANOVA followed by Tukey's post-hoc test. A *P*-value of less than 0.05 was considered statistically significant.

## Data Availability

The datasets generated during and/or analysed during the current study are available from the corresponding author on reasonable request.
